# HIV-1 Subtype C Mosaic Gag Expressed by BCG and MVA Elicits Persistent Effector T Cell Responses in a Prime-Boost Regimen in Mice

**DOI:** 10.1371/journal.pone.0159141

**Published:** 2016-07-18

**Authors:** Tsungai Ivai Jongwe, Ros Chapman, Nicola Douglass, Shivan Chetty, Gerald Chege, Anna-Lise Williamson

**Affiliations:** 1 Institute of Infectious Disease and Molecular Medicine and Division of Medical Virology, Department of Pathology, Faculty of Health Sciences, University of Cape Town, Cape Town, South Africa; 2 National Health Laboratory Services, Groote Schuur Hospital, Cape Town, South Africa; Commissariat a l'Energie Atomique(cea), FRANCE

## Abstract

Over 90% of HIV/AIDS positive individuals in sub-Saharan Africa are infected with highly heterogeneous HIV-1 subtype C (HIV-1C) viruses. One of the best ways to reduce the burden of this disease is the development of an affordable and effective prophylactic vaccine. Mosaic immunogens are computationally designed to overcome the hurdle of HIV diversity by maximizing the expression of potential T cell epitopes. *Mycobacterium bovis* BCG Δ*panCD* auxotroph and modified vaccinia Ankara (MVA) vaccines expressing HIV-1C mosaic Gag (Gag^M^) were tested in a prime-boost regimen to demonstrate immunogenicity in a mouse study. The BCG-Gag^M^ vaccine was stable and persisted 11.5 weeks post vaccination in BALB/c mice. Priming with BCG-Gag^M^ and boosting with MVA-Gag^M^ elicited higher Gag-specific IFN-γ ELISPOT responses than the BCG-Gag^M^ only and MVA-Gag^M^ only homologous vaccination regimens. The heterologous vaccination also generated a more balanced and persistent CD4^+^ and CD8^+^ T cell Gag-specific IFN-γ ELISPOT response with a predominant effector memory phenotype. A Th1 bias was induced by the vaccines as determined by the predominant secretion of IFN-γ, TNF-α, and IL-2. This study shows that a low dose of MVA (10^4^ pfu) can effectively boost a BCG prime expressing the same mosaic immunogen, generating strong, cellular immune responses against Gag in mice. Our data warrants further evaluation in non-human primates. A low dose vaccine would be an advantage in the resource limited countries of sub-Saharan Africa and India (where the predominating virus is HIV-1 subtype C).

## Introduction

HIV-1 subtype C (HIV-1C) is the most prevalent subtype in the world, accounting for over 50% of all global infections [1,2]. It is the dominant subtype in southern Africa where it accounts for more than 95% of all HIV-1 infections in the region [3–6] and in India (which accounts for 6% of the HIV-1 global prevalence of HIV-1C) [2,7]. In addition, HIV-1C infections have been shown to be on the increase in Wales, England [8], and South America [9,10].

A prophylactic HIV-1 vaccine is thought to be the most effective means of controlling the spread of this epidemic. Furthermore, there is general agreement that a successful vaccine will need to induce strong humoral and cellular immune responses [11–13]. Developing such a vaccine is particularly challenging in the face of the diversity of HIV-1. In HIV-infected elite controllers and long-term non-progressors lower viral loads are significantly correlated with Gag-specific CD4^+^ and CD8^+^ T cell responses [14–16]. Therefore HIV-1 Gag is a critical immunogen for inclusion in the T-cell inducing component of an HIV-1 vaccine. One of the methods used to address the diversity of HIV-1, is the generation of mosaic and conserved immunogens which can be designed computationally (reviewed in [17,18]). Mosaic immunogens are optimised *in silico* to increase the coverage of both CD8^+^ and CD4^+^ T cell epitopes from natural sequences with the hope of reducing the HIV-1 escape pathways [19–26]. This strategy provides a higher level of diversity coverage when compared to natural sequence vaccine candidates.

In this paper *Mycobacterium bovis* bacillus Calmette-Guérin (BCG) and modified vaccinia Ankara (MVA) are investigated as HIV vaccine vectors to deliver an HIV-1C Gag mosaic antigen targeted at inducing T cell responses. Since its development as a safe tuberculosis vaccine, BCG has been administered to over 4 billion infants and is accessible to 80% of infants globally [27–29]. The results from animal model studies indicate that recombinant BCG (rBCG) can be used as a vaccine vector that can induce both humoral and antigen-specific cellular immune responses to HIV-1 (reviewed in [30,31]). In the event that HIV-1-infected and immunocompromised individuals unintentionally get enrolled in future BCG-vectored HIV-1 vaccine campaigns, it is essential to use the safer BCG strains as vectors [32]. Strategies have been developed for the production of safer BCG strains, in particular auxotrophic BCG strains. These strains have mutations in genes required for the production of essential growth compounds, and so cannot replicate unless supplemented with the necessary growth compounds [33–36]. We used a Δ*panCD* auxotroph which is unable to synthesize pantothenic acid, a key precursor of coenzyme A, which is essential for several mycobacterial intracellular processes [32,37,38]. Furthermore, since safer strains of BCG are being developed against childhood TB, it is important to use these strains as HIV-1 vaccine vectors for use as dual vaccines.

The only effective HIV vaccine trial (RV144) tested a combination of a canary poxvirus vector and protein as candidate vaccines encouraging further exploration of poxviruses as HIV vaccine vectors. MVA is a highly attenuated strain of the smallpox virus vaccine, vaccinia virus (VACV), that is unable to complete its replication cycle in human cells due to large deletions [39]. Furthermore, deletion of immune-modulatory genes results in a rapid local immune response at the point of infection [39,40]. MVA has adjuvant properties and potential to induce long-lasting immunity. However, viral and foreign genes can still be efficiently expressed. Since smallpox was eradicated and vaccination ceased in 1979, people up to 37 years of age would not have been exposed to either *Variola virus* or VACV and therefore would not have been immunized with MVA. MVA provides the safety of an attenuated or killed virus vaccine, yet provides the immunogenicity of a live virus vaccine with its ability to express foreign antigen. Added to their safety, both rBCG and rMVA are affordable to produce [41–43].

In an effort to make affordable vaccines suitable for the regions most affected by HIV-1, we developed stable HIV-1 vaccines—based on BCG and MVA vectors—expressing an HIV-1C mosaic Gag immunogen and assessed the vaccine immunogenicity in mice.

## Materials and Methods

### Construction of rBCG

The pantothenic acid auxotroph strain derived from BCG Pasteur, BCGΔ*panCD*, was kindly provided by Professor William R Jacobs and cultured as previously described [37].

The BCG shuttle vector pTJBCG3 was constructed as follows: the full length HIV-1C mosaic *gag* gene (*gag*^*M*^ [19]) was codon optimised for use in BCG and cloned into the *Cla*I and *Hpa*I sites of pHS400 [37]. A control plasmid that had no insert in it (pEM19) was included in the study. To generate plasmid pEM19, pHS400 was digested with *Sna*BI and *Hpa*I, to remove *gag*^*M*^, and religated.

Recombinant BCGΔ*panCD* vaccine stocks resuspended in 8.5% w/v NaCl; 10%glycerol; 10% Tyloxapol were made as previously described [37] and stored at -80°C until required.

### *In Vitro* and *In Vivo* Genetic Integrity of rBCG Vaccine Stocks

Crude cell lysates prepared from BCGΔ*panCD* vaccine stocks were electroporated into *E*. *coli* DH5α electro-competent cells which were plated on Luria agar [44]. To determine the *in vitro* plasmid stability, single colonies from the electroporated *E*. *coli* cells were used to inoculate liquid media, plasmid DNA was isolated and mapped using restriction enzyme digestion. *In vivo* shuttle vector integrity was determined on spleens and lymph nodes isolated from mice 11.5 weeks after vaccination by polymerase chain reaction (PCR) using crude cell lysates of BCG colonies obtained after plating homogenised splenocytes and lymph nodes from previously vaccinated mice on Middlebrook 7H10 agar. The PCR components were 5 μl of BCG crude cell lysate, 25μl PCR ImmoMix Red (Bioline, USA), 10μM each primer (pCB119F: 5'–CAT ATG AAG CGT GGA CTG AC– 3' and pEMRev: 5'–AGC AGA CAG TTT TAT TGT TC—3') and 10μl distilled water. The PCR reaction conditions were initial denaturation at 95°C for 10 minutes, followed by 30 cycles of DNA denaturation at 95°C for 30 seconds, annealing of primers at 56°C for 30 seconds, and DNA extension at 72°C for one minute with a 5 second increment per cycle. A final extension step at 72°C for 4 minutes completed the reaction. PCR products were confirmed for lack of mutations by sequence analysis.

### Construction of Recombinant MVA (rMVA)

**Fig 1:**
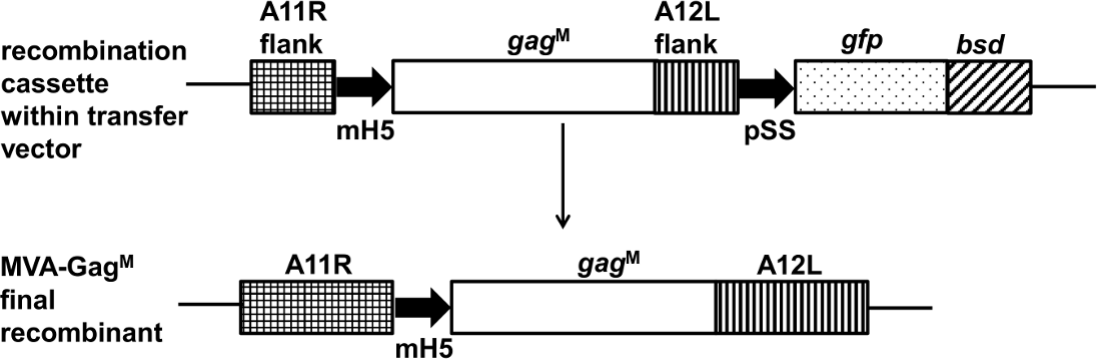
Recombinant MVA construction. The transfer vector pTJMVA2 was designed to insert the *gag*^*M*^ gene under the transcriptional control of the mH5 promoter, between the MVA A11R and A12L ORFs. The selection (*bsd*) and marker (*gfp*) genes were expressed as a fused protein under the transcriptional control of the pSS MVA synthetic promoter.

The transfer vector, pTJMVA2, was designed to insert the *gag*^M^ gene, under the control of the VACV mH5 promoter, between the A11R and A12L transcriptionally convergent open reading frames (ORFs) (Fig 1). The genes for green fluorescent protein (*gfp*) and blasticidin resistance (*bsd*) were used as marker and selection genes respectively and expressed as a fusion protein under the transcriptional control of the pSS promoter. The *gfp-bsd* ORF was placed outside of the A12L flank, so that it could be recombined out at a later stage and non-fluorescing foci could be screened for the final recombinant containing *gag*^M^ alone between the A11R and A12L ORFs.

Adherent BHK cell monolayers were infected with wild type (wt) MVA (multiplicity of infection (MOI) = 0.1) diluted in DMEM-0 (DMEM supplemented with 1000U/ml penicillin (Lonza, Belgium), 1000U/ml streptomycin (Lonza, Belgium), and 10μg/ml fungin (Invivogen, USA)) and transfected 2 hours later with a 1:1 ratio of 4μg of the recombinant plasmid DNA, pTJMVA2: 4μl X-treme Gene HP® transfection reagent (Roche, Switzerland) according to the manufacturer’s instructions. Cells were incubated at 37°C in a 5% CO_2_ humidified incubator for 48 hours. Total virus was harvested and used to re-infect a fresh BHK monolayer. Virus was passaged 4 times in the presence of blasticidin and an intermediate rMVA expressing GFP was purified. Virus was then passaged in the absence of blasticidin and non-fluorescing foci were plaque purified 7 times. PCR was carried out to screen for the presence of the intermediate and final MVA recombinants using cell lysates of infected cells as template. The presence of the intermediate recombinant MVA was confirmed by PCR amplification using the primers A11R*for* (5'-ACAAACACCATCCTTGGGAGTA-3') and gfp*rev* (5'-AAAGTTCTTCACCCTTAGACGCC-3') that bind to A11R and *gfp* respectively, or colE1*for* (5'-GCGTGAGCTATGAGAAAGCGCCAAAT-3') and A12L*rev* (5'-CGGTGGAGATGCAGCCGTCAA-3') that bind to the *E*. *coli* ORI and A12L respectively. The presence of the final recombinant was confirmed using a *gag*-specific primer in combination with a primer which bound on either side of the A12L or A11R flanking site in the MVA genome (gag*for*: 5'-CCCTAGAAAGAAAGGCTGCTGGAA-3' and A12L*rev*: 5'-AATCGGTGGAGATGCAGCCGTCAA-3' or A11R*for*: 5'-ACAAACACCATCCTTGGGAGTATTC-3' and gag*rev*: 5'-TTTCCGCCAGGCCTCAGT-3') and using primers A11R*for* and A12L*rev* alone. Cell lysates for PCR were obtained using a method described by David Tscharke (personal communication). Briefly, when plaques were visible, the cells were gently washed with 1ml PBS/well. A volume of 250μl 1 X PCR buffer (10mM TrisCl, 2.5mM MgCl_2_, 50mM KCl, pH 8.3) with proteinase K (10μg/ml; Sigma-Aldrich, USA) was added to the cells, incubated at -80°C until frozen, and then thawed at 37°C. Cell lysates were incubated at 56°C for 20 minutes, and then at 85°C for 10 minutes. Cell debris was removed by centrifugation for 10 minutes at 2000rpm (Eppendorf Centrifuge 5417C, Germany). A 5–10μl aliquot of the supernatant was used for PCR using an ImmoMix Red PCR mix (Bioline, USA) according to the manufacturer’s instructions. The PCR product for the final recombinant was confirmed by sequencing using the ABI Prism® BigDye™ Terminator Cycle Sequencing kit (Applied Biosystems, USA) according to the manufacturer’s instructions. This was done as a service offered by the Central Analytical Facilities laboratory (Stellenbosch University, South Africa). MVA-Gag^M^ virus stock was grown in BHK cells and purified on a 36% sucrose cushion (36% in PBS) at 4°C and 15 000rpm (Sorvall RCSC Plus, USA) for an hour. The virus stock was further purified on a sucrose-dextran gradient (36% sucrose; 10% dextran) under the same conditions described above, and stored in PBS at −80°C until required. MVA-Gag^M^ virus titration was done on BHK-21 cells as previously described by Chapman *et al*., 2012 [37]. Western blot analysis to determine Gag^M^ protein expression was carried out as previously described [42,45].

### Mice Vaccinations

Groups of four to five 6–8 weeks old female BALB/c mice were used for each experiment. The BCG vaccine was administered intraperitoneally at a dose of 2 x 10^7^cfu per mouse. MVA vaccines were administered bilaterally into the tibialis muscle of each hind leg (50μl each) at a total dose of 10^2^, 10^4^ or 10^6^ pfu MVA. Mice were sacrificed by cervical dislocation. The vaccination schedule (see Table 1) and all the animal procedures were approved by the University of Cape Town Animal Research Ethics Committee (reference UCT AEC 12–059) and performed by a trained animal technologist.

**Table 1:** Mouse immunization regimen.

Group	Prime	Boost	Sacrifice
**1**	**Day 0:** 2x10^7^ cfu BCG-Gag^M^	**Day 70:** 10^2^ pfu MVA-Gag^M^	**Day 82**
**2**	**Day 0:** 2x10^7^ cfu BCG-Gag^M^	**Day 70:** 10^4^ pfu MVA-Gag^M^	**Day 82**
**3**	**Day 0:** 2x10^7^ cfu BCG-Gag^M^	**Day 70:** 10^6^ pfu MVA-Gag^M^	**Day 82**
**4**	**Day 0:** 2x10^7^ cfu BCG^E^	**Day 70:** 10^2^ pfu MVA-Gag^M^	**Day 82**
**5**	**Day 0:** 2x10^7^ cfu BCG^E^	**Day 70:** 10^4^ pfu MVA-Gag^M^	**Day 82**
**6**	**Day 0:** 2x10^7^ cfu BCG^E^	**Day 70:** 10^6^ pfu MVA-Gag^M^	**Day 82**
**16**	**Day 0:** 2x10^7^ cfu BCG-Gag^M^	**Day 70:** 2x10^7^ cfu BCG-Gag^M^	**Day 82**
**17**		**Day 0:** 10^4^ pfu MVA-Gag^M^	**Day 12**
**18**	**Day 0:** 10^4^ pfu MVA-Gag^M^	**Day 28:** 10^4^ pfu MVA-Gag^M^	**Day 40**

### Isolation of Splenocytes and Lymph Nodes

At the experimental endpoint, spleens and mesenteric lymph nodes were harvested. Organs of the same type in each group were pooled before processing. A single cell suspension from the spleens was prepared as previously described [37,46] for use in the IFN-γ ELISPOT assay, intracellular cytokine staining and for the cytometric bead array assay. Mesenteric lymph nodes and left over splenocytes from the BCG vaccinations were stored at -80°C in BCG resuspension buffer (8.5% w/v NaCl, 10% glycerol, 10% tyloxapol) until required for evaluating the integrity of the shuttle vectors.

### Immunogenicity Assays to Evaluate Mosaic Vaccines

IFN-γ ELISPOT and cytometric bead array (CBA) assays were carried out as previously described [37] and intracellular cytokine staining and staining of cell surface molecules was carried out as previously described by Burgers et al., 2006 [47]. The following antibodies were used; anti-CD3^+^ Alexa 700, anti-CD4^+^ PE-Cy7, anti-CD8^+^ APC-Cy7, anti-CD62L APC, and anti-CD44 FITC). The cytokine antibodies were all PE-conjugated (0.2μg anti-TNF-PE, 0.06 μg anti-IL-2-PE, 0.06 μg anti-IFN-γ-PE) and were obtained from BD biosciences. The anti-CD4^+^ PE-Cy7, anti-CD8^+^ APC-Cy7 were obtained from BD biosciences. All the other antibodies were obtained from eBioscience.

### Statistical Analysis

Data was statistically analysed using Prism version 5.0 (Graphpad Software, San Diego, CA). The *t* test for independent unpaired parametric comparisons was applied to assess the level of significance of comparisons between means. All tests were two-tailed. *P* values ≤ 0.05 were considered significant. The false discovery rate (FDR) step-down procedure described in the paper by Columb & Sagadai [48] was used to correct for multiple comparisons.

## Results

### Determination of rBCG Vaccine Stability

**Fig 2:**
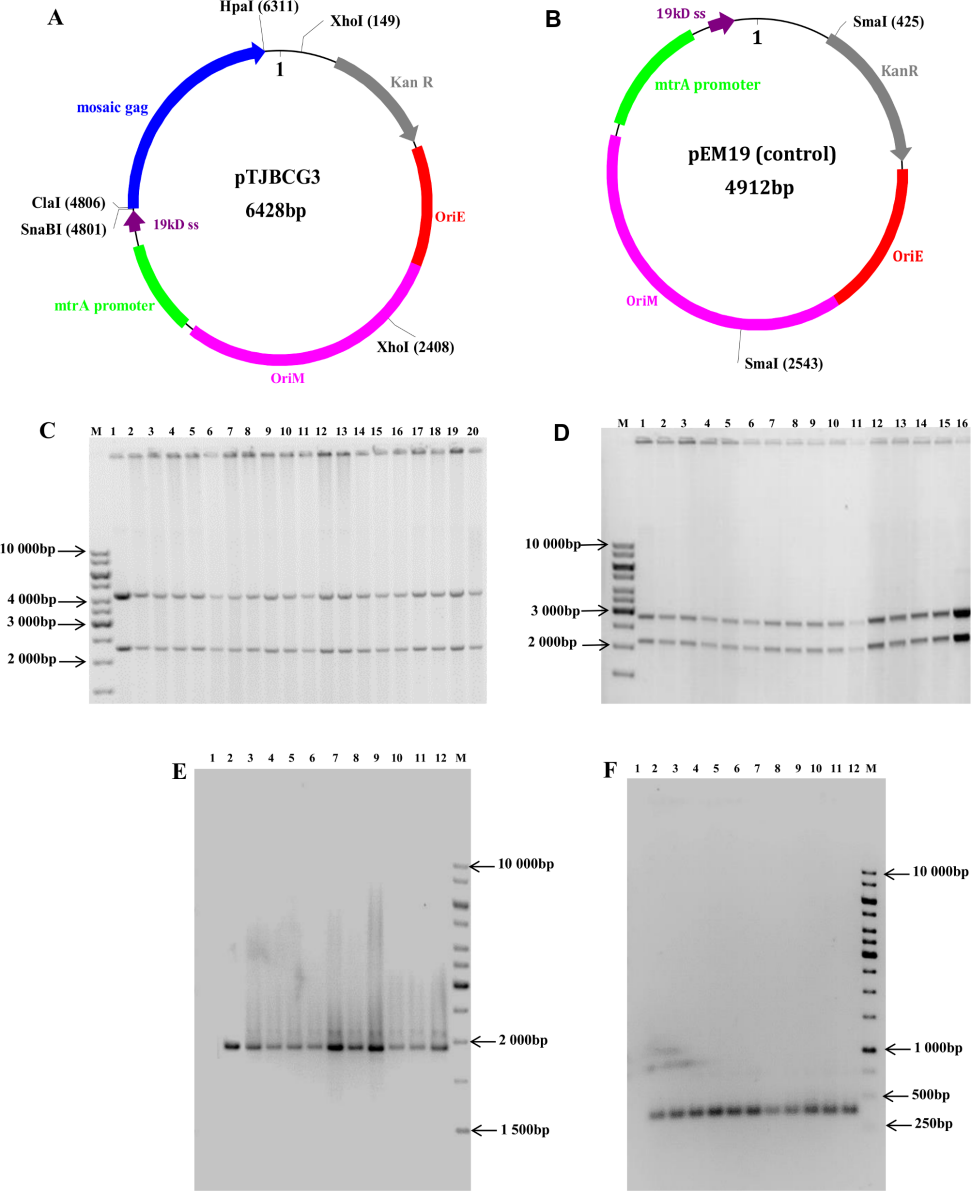
Schematic representation of the BCG shuttle vectors and the determination of their genetic integrity before and after vaccination. Schematic representation of (**A**) pTJBCG3, which was used to make the BCG-Gag^M^ vaccine, (**B**) pEM19 which was used to make the BCG^E^ vaccine. Restriction sites used for cloning and restriction mapping analysis are indicated in black bold type. OriE–*E*. *coli* origin of replication; OriM–mycobacterial origin of replication, 19kD ss– 19kD signal sequence; KanR–kanamycin resistance gene. (**C**) pTJBCG3 digested with *Xho*I. Lane 1 is a positive control of pTJBCG3 DNA prior to transformation into BCGΔ*panCD*. Lanes 2–21 contain pTJBCG3 DNA obtained from recombinant BCG-Gag^M^ vaccine stocks. (**D**) pEM19 digested with *Sma*I. Lanes 1–15 contain pEM19 plasmid DNA isolated from recombinant BCG^E^ vaccine stocks, and lane 16 pEM19 plasmid DNA isolated prior to transformation into BCGΔ*panCD* (positive control). PCR amplification of DNA from rBCG obtained from the spleens and lymph nodes of mice vaccinated with BCG-Gag^M^ (Group 2) (**E**) or BCG^E^ (Group 5) (**F**) 11.5 weeks post vaccination. Lane 1 –negative control; Lane 2 –positive control; Lane 3–12 are PCR products from rBCG isolated from homogenised spleen (3–7) or lymph nodes (8–12). Lanes M in **C**—**F** contain the molecular weight marker O’GeneRuler^TM^ 1kb DNA ladder.

As the stability of rBCG can be compromised *in vitro* and *in vivo* (reviewed by Chapman *et al*., 2010; [49]), the genetic integrity of the rBCGΔ*panCD* vaccine stocks was assessed by restriction enzyme mapping of the shuttle vectors using *Xho*I (pTJBCG3) and *Sma*I (pEM19) (Fig 2A–2D). A single batch of each vaccine stock was tested and the same batch was then used to inoculate mice. pTJBCG3 and pEM19 plasmid DNA isolated from vaccine stocks of BCG-Gag^M^ and BCG^E^ respectively gave DNA fragments of the expected sizes (Fig 2C and 2D).

The genetic integrity of the BCG shuttle vectors *in vivo* was determined by PCR analysis of plasmid DNA extracted from rBCG isolated from the spleens and lymph nodes of mice 11.5 weeks post vaccination. PCR products of the expected sizes of 1869bp and 344bp were obtained from pTJBCG3 and pEM19 respectively (Fig 2E and 2F). Eight of the PCR products were also sequenced to confirm genetic integrity and no mutations were observed (data not shown).

### Generation of a Recombinant MVA Expressing Gag^M^

**Fig 3:**
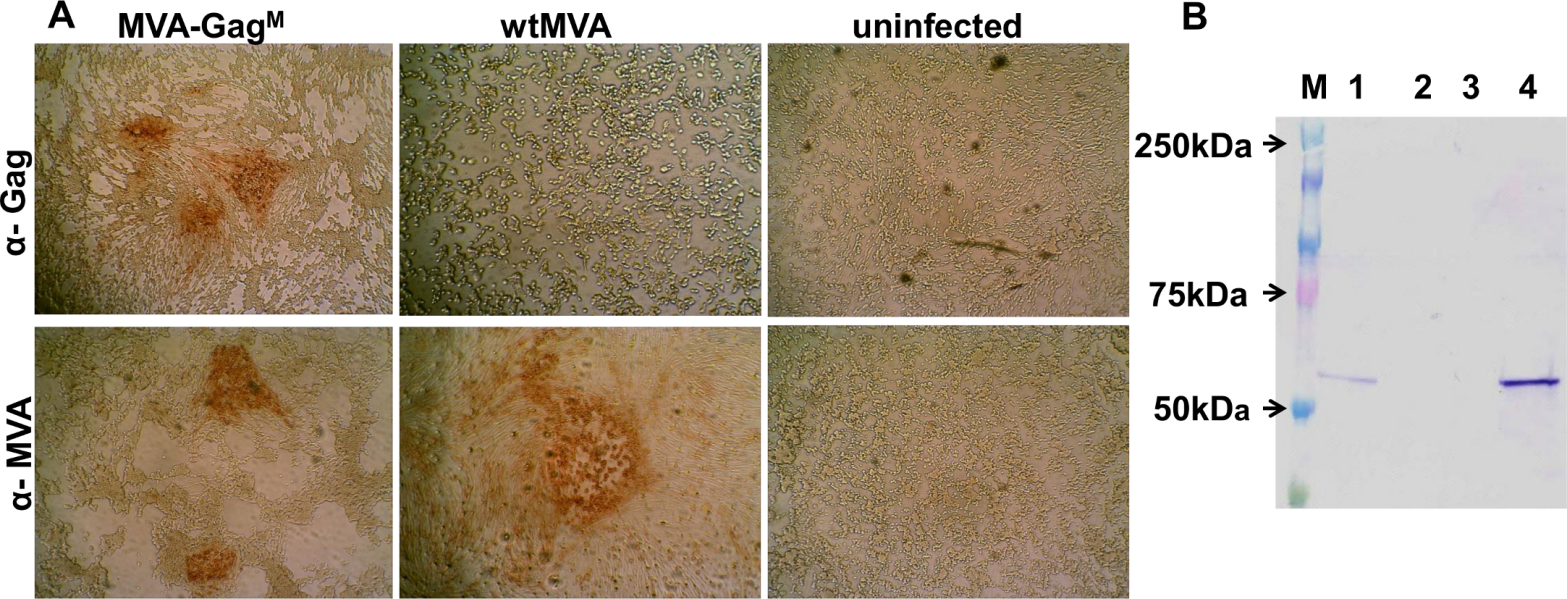
*In vitro* expression of Gag by BHK-21 cells infected with MVA-Gag^M^. (**A**) BHK-21 cells were infected with MVA-Gag^M^, wild type MVA (wtMVA), or left uninfected for 72 hours. HIV-1 Gag was detected with anti-Gag antibody ARP432 (α-Gag; top panels) as well as an anti-VACV antibody (α-MVA; bottom panels), followed by an anti-rabbit HRP-conjugated antibody. (**B**) Cell lysates were prepared from BHK-21 cells infected with MVA-Gag^M^ (lane 4), wild type MVA (lane 3), or left uninfected (lane 2). BHK cells transfected with a plasmid known to express full length Gag was used as a positive control (lane 1). Western blots were probed with a rabbit anti-HIV-1-p24 Gag antibody (ARP432), followed by an anti-rabbit antibody conjugated to alkaline phosphatase (Sigma-Aldrich, USA). A Precision Plus Protein Kaleidoscope pre-stained standard (lane M; Biorad, USA) was used and the sizes are indicated on the left.

A recombinant MVA was constructed with HIV-1C *gag*^M^ inserted between ORFs A11R and A12L and under the control of the vaccinia virus mH5 promoter. Gag^M^ expression was confirmed by immunostaining using anti-HIV-1 p24 Gag antibody (ARP432) (Fig 3A). Uninfected cells and cells infected with wtMVA were used as negative controls. *In vitro* expression of the Gag^M^ protein in the vaccine stocks was also confirmed by Western blot analysis following SDS PAGE. Cell lysates derived from BHK-21 cells infected with MVA-Gag^M^, when probed with a Gag-specific antibody, showed expression of a protein of the correct size of 55kD (Fig 3B; Lane 4). Lysates from uninfected cells were used as a negative control and a BHK cell lysate transfected with a plasmid known to express full length Gag was used as a positive control (Fig 3B; Lane 1) [47].

### Determination of the Optimal Dose of MVA-Gag^M^ to Boost a BCG-Gag^M^ Prime

**Fig 4:**
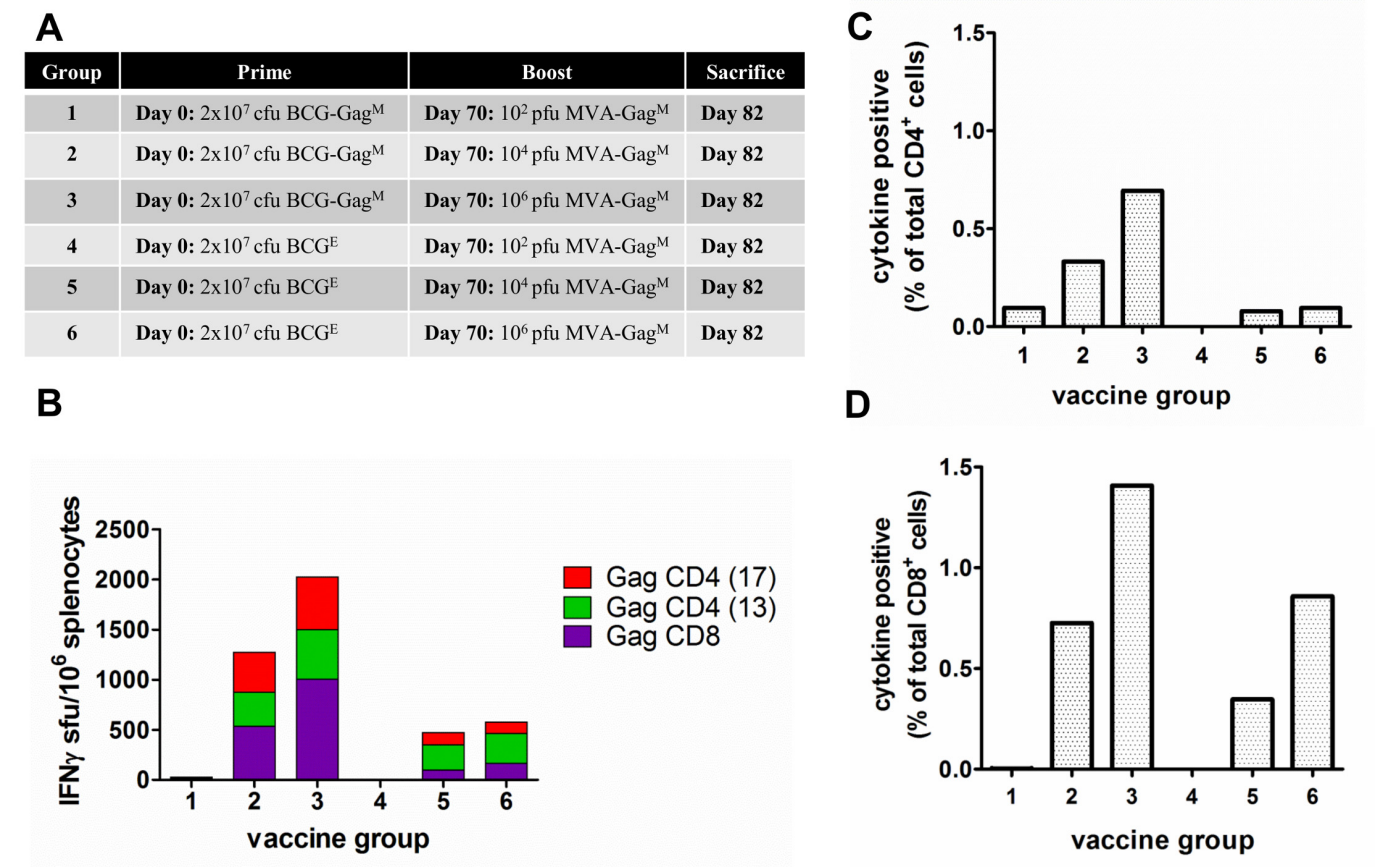
Determination of the optimal dosage of MVA-Gag^M^ to boost a BCG-Gag^M^ prime. (**A)** Mice were primed on day 0 with 2 x 10^7^ cfu BCG-Gag^M^ (Group 1–3) or BCG^E^ (Group 4–6) and boosted on day 70 with 10^2^ (Group 1 and 4), 10^4^ (Group 2 and 5), or 10^6^ (Group 3 and 6) pfu MVA-Gag^M^. (**B**) Cumulative IFN-γ ELISPOT CD8^+^ and CD4^+^ responses of vaccinated mice to HIV-1 Gag peptides. The ELISPOT assay was carried out using three Gag-specific peptides for stimulation of pooled splenocytes that were isolated 12 days post the MVA-Gag^M^ boost. Bars represent the magnitude of net responses to individual peptides, expressed as sfu/10^6^ splenocytes after subtracting the background. (**C**) and (**D**) Total frequency of T cells producing IFN-γ, IL-2, and/or TNF-α, after subtracting the background, in response to HIV-1 Gag peptide stimulation following a rBCG prime and an MVA-Gag^M^ boost at different doses. Cells were positive for cytokine production if the proportion was ≥0.05% after subtracting the background. These results are from a single experiment using pooled splenocytes.

To determine the optimal MVA dose required to effectively boost the BCG prime, mice were primed with 2 x 10^7^ cfu of either the recombinant BCG-Gag^M^ or the control BCG^E^ and boosted on day 70 with 10^2^, 10^4^, or 10^6^ pfu of MVA-Gag^M^ (see table insert in Fig 4). The BCG vaccine priming dose of 2 x 10^7^ cfu was previously determined as optimal in our lab (Dr Ros Chapman, personal communication).

Priming with the BCG-Gag^M^ or BCG^E^ and boosting with 10^2^ pfu MVA-Gag^M^ elicited no detectable Gag-specific IFN-γ ELISPOT responses in mice (Fig 4B; Groups 1 and 4 respectively). However, high cumulative HIV-1 Gag-specific IFN-γ ELISPOT responses were induced in mice primed with BCG-Gag^M^ and boosted with 10^4^ pfu (1273 sfu/10^6^ splenocytes) and 10^6^ pfu (2023 sfu/10^6^ splenocytes) MVA-Gag^M^. These responses targeted both Gag CD4 and CD8 peptides in almost equal magnitudes (Fig 4B, Groups 2 and 3 respectively). The magnitudes of these responses were 3.2 and 4.1 fold higher, than those induced in mice primed with the control BCG (BCG^E^) and boosted with a 10^4^ or 10^6^ pfu of MVA-Gag^M^ respectively (Groups 5 & 6). Thus, MVA-Gag^M^ efficiently boosts a BCG-Gag^M^ prime at a dose of 10^4^ pfu or 10^6^ pfu but not at a dose of 10^2^ pfu.

To further characterise the immune responses induced by boosting BALB/c mice with different doses of MVA-Gag^M^ following a BCG prime, intra-cellular cytokine staining followed by flow cytometry was carried out to determine the combined frequency of IFN-γ-, TNF-α-, and IL-2- producing cells (Fig 4C and 4D).

Priming with BCG-Gag^M^ or with the control BCG^E^, and boosting with 10^2^ pfu MVA-Gag^M^ did not elicit any detectable HIV-1 Gag-specific cytokine-producing CD8^+^ T cells (Fig 4D, Groups 1 and 4). Mice primed with BCG-Gag^M^ and boosted with 10^4^ pfu (Group 2) or 10^6^ pfu MVA-Gag^M^ (Group 3) elicited almost double the frequency of HIV-1 Gag-specific cytokine-producing CD8^+^ T cells as compared to control-primed mice that were similarly boosted (Groups 5 &6), suggesting an effective BCG-Gag^M^ prime.

### Immune Responses in BALB/c Mice Elicited by BCG Prime-MVA Boost Vaccines Expressing a Gag^M^ Immunogen

While the greatest cumulative immune response to the Gag peptides was detected from mice boosted with 10^6^ pfu MVA-Gag^M^ (Fig 4B; Group 3), the amount of background responses in the IFN-γ ELISPOT assay were unacceptably high (up to 420 sfu/10^6^ splenocytes). An MVA-Gag^M^ boost of 10^4^ pfu for rBCGΔ*panCD*-primed mice was therefore chosen as the optimal dose to compare immune responses to different rBCGΔ*panCD* prime vaccinations. As controls, mice vaccinated with a single dose of MVA-Gag^M^ as well as those vaccinated with two homologous doses of BCG-Gag^M^ or MVA-Gag^M^ vaccines were included. The immunisation schedules were chosen to give the peak immune response for each vaccine vector or combinations of vaccine vectors. Data from our group (unpublished) has indicated immune responses are improved if the MVA boost is given at 10 weeks rather than 4 weeks post the BCG prime.

**Fig 5:**
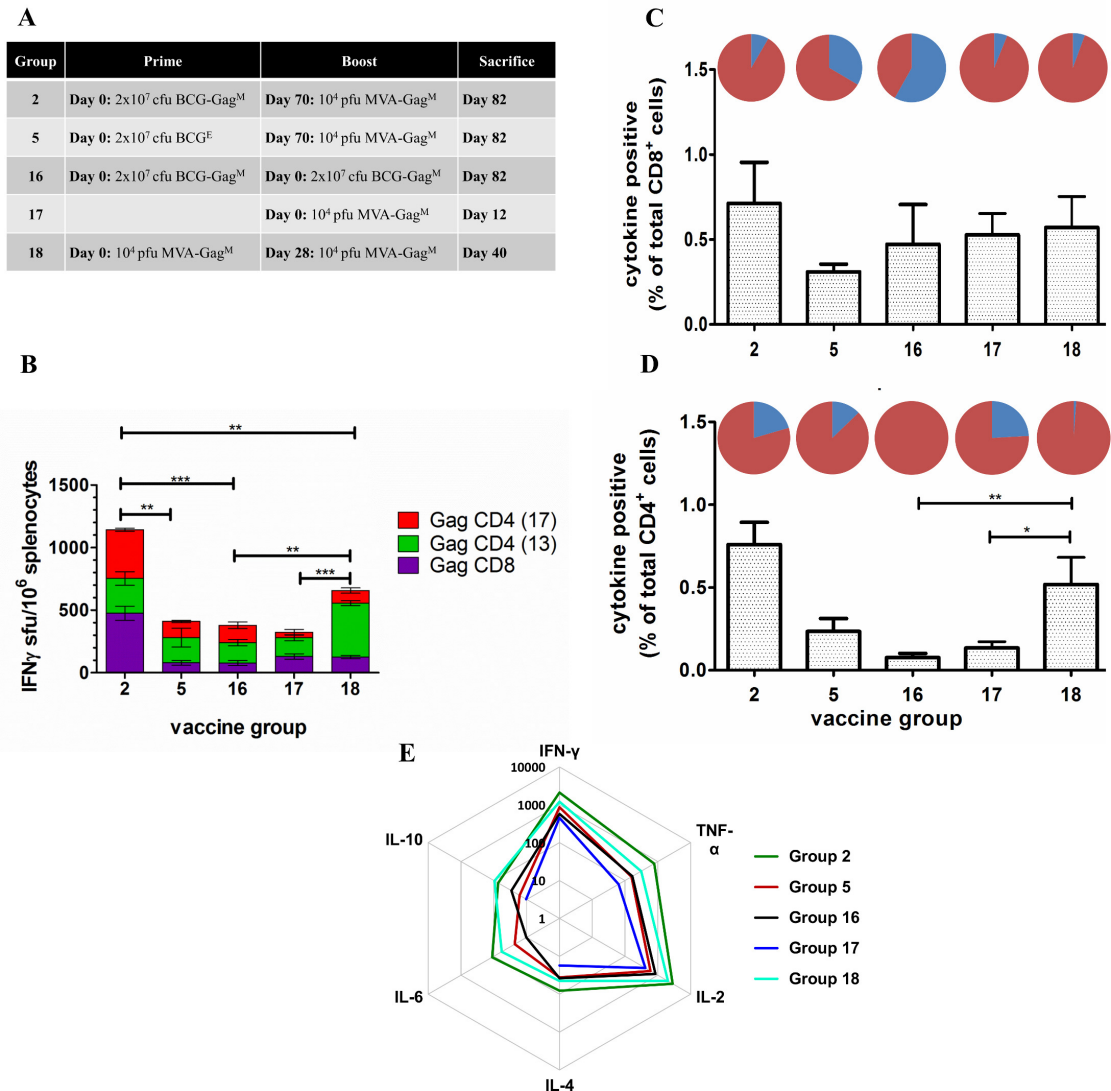
Evaluation of a BCG-Gag^M^ prime/ MVA-Gag^M^ boost in BALB/c mice. (**A**) Vaccination schedule used. (**B**) Cumulative IFN-γ ELISPOT CD8^+^ and CD4^+^ responses of vaccinated mice to HIV-1 Gag peptides. The ELISPOT assay was done in triplicate using pooled spleens on the day of sacrifice using three Gag-specific peptides for stimulation. Bars are the mean and standard deviation of the mean responses for the indicated individual peptides from 3 independent experiments. Responses are expressed as sfu/10^6^ splenocytes after background subtraction. Horizontal bars with asterisks indicate statistical significance of the mean responses between the indicated groups. **p<0.01, ***p<0.001; Student t-test of unpaired data followed by FDR for multiple comparisons. (**C**) and (**D**) Total frequency of T cells producing IFN-γ, IL-2, and/or TNF-α in response to HIV-1 Gag peptide stimulation. Cell surface staining and flow cytometry were carried out in duplicate on pooled spleens per group using three Gag-specific peptides for stimulation. The memory distribution of the cytokine producing T- cells in the central and effector memory compartment (T_CM_ and T_EM_) are represented as pie charts above each corresponding bar per group. Cells were positive for cytokine production if the proportion was ≥ 0.05% after subtracting the background. The cellular phenotype was positive if there were ≥ 10 cells per test. (**E**) The levels of cytokines in the culture supernatants were quantified using a Th1/Th2 cytokine bead array assay followed by flow cytometry. The recorded results were obtained after subtracting the background. The distance from the centre of the plot indicates a log_10_-fold change (ranging from 1 to 10 000) and cytokine levels were expressed as pg/ml.

Mean cumulative responses for Group 2 mice (BCG-Gag^M^/MVA-Gag^M^) reached a magnitude of 1143 ± 117 sfu/10^6^ splenocytes with almost equally balanced responses targeted to Gag CD8 (475 ± 55 sfu/10^6^ splenocytes) and Gag CD4 (668 ± 32.7 sfu/10^6^ splenocytes; Fig 5B) peptides. There was a 2.8-fold difference between the magnitudes of cumulative responses of mice primed with BCG-Gag^M^ (Group 2) and those that received a control BCG^E^ prime (Group 5–410 ± 98 sfu/10^6^ splenocytes). Thus, BCG-Gag^M^ efficiently primes the adaptive immune system.

Mean cumulative IFN-γ ELISPOT HIV-1 Gag responses of mice vaccinated with the BCG-Gag^M^/MVA-Gag^M^ heterologous prime-boost regimen (Group 2) were 3- and 1.7-fold greater than the BCG-Gag^M^/BCG-Gag^M^ homologous prime-boost (Group 16; 380 ± 64.7 sfu/10^6^ splenocytes) and MVA-Gag^M^/MVA-Gag^M^ homologous prime-boost; 656.7 ± 8.5 sfu/10^6^ (Group 18) mice respectively. The BCG-Gag^M^/MVA-Gag^M^ heterologous prime-boost was therefore significantly more efficient in generating an immune response than the BCG-Gag^M^/BCG-Gag^M^ (p<0.001) and MVA-Gag^M^/MVA-Gag^M^ (p<0.01) homologous prime-boost vaccinations. Interestingly, responses to the CD8 Gag peptide were similar for Groups 17 (MVA-Gag^M^) and 18 (MVA-Gag^M^/ MVA-Gag^M^; Fig 5B). The second MVA-Gag^M^ vaccination, however boosted CD4^+^ T cell responses to Gag (Fig 5B).

As shown in Fig 5C and 5D, the BCG-Gag^M^/MVA-Gag^M^ heterologous prime-boost regimen (Group 2) resulted in CD8^+^ T cells with a higher frequency of effector memory phenotype (91.6%) than those in the control group (Group 5–66.5%). A BCG-Gag^M^ homologous prime-boost resulted in cytokine positive CD8^+^ T cells that were predominantly of a central memory phenotype (58%; Group 16). There were fewer cytokine-producing CD4^+^ T cells than there were cytokine-producing CD8^+^ T cells for all vaccination regimens except for the Group 18 mice that received an MVA-Gag^M^ homologous prime-boost vaccination (Fig 5C and 5D). A single MVA-Gag^M^ vaccination resulted in cytokine-positive CD4^+^ T cells with a predominant effector memory phenotype (Group 17–76%), and a homologous boost increased the proportion of effector memory CD4^+^ T cells to 99% (Group 18). Cytokine-positive CD4^+^ T cells following a BCG-Gag^M^ homologous prime-boost all had an effector memory phenotype (Group 16).

To assess the Th1/Th2 bias of the immune response to the vaccines used, Th1 and Th2 cytokines were quantified in culture media of splenocytes stimulated with Gag CD4 and CD8 peptides in a cytokine bead array assay (Fig 5E). As anticipated, IFN-γ, TNF-α, and IL-2 (Th1 cytokines) had the highest cumulative levels in all groups of mice. IFN-γ, TNF-α, and IL-2 were 2.4-, 4.9-, and 4.7-fold higher, respectively, in Group 2 (BCG-Gag^M^/MVA-Gag^M^) compared to Group 5 mice (BCG^E^/MVA-Gag^M^) confirming an efficient prime with the BCG-Gag^M^ vaccine. The MVA-Gag^M^ vaccine also potently boosted the BCG-Gag^M^ prime. Cumulative IFN-γ, TNF-α, and IL-2 levels were higher in mice that received the heterologous BCG-Gag^M^/MVA-Gag^M^ prime-boost vaccination (Group 2) compared to Group 16 mice that received a BCG-Gag^M^/BCG-Gag^M^ homologous vaccination. Splenocytes from the heterologous prime-boost vaccination (Group 2) produced high levels of IFN-γ, TNF-α, and IL-2 compared to any of the homologous prime-boost vaccinations (Group 16 and 18; BCG-Gag^M^/BCG-Gag^M^ and MVA-Gag^M^/MVA-Gag^M^ respectively).

## Discussion

In this study, rBCGΔ*panCD* and rMVA vaccines expressing an HIV-1C mosaic Gag (BCG-Gag^M^ and MVA-Gag^M^, respectively) were made and evaluated in mice. Studies done by others have shown HIV-1 Group M mosaic vaccines to induce broader T cell and higher magnitude responses than vaccines expressing antigens derived from natural or consensus HIV-1 sequences [21,26,50–52]. Here we have shown BCG-Gag^M^ and MVA-Gag^M^ vaccines expressing HIV-1C mosaic Gag to be immunogenic in mice, particularly when administered as a heterologous prime-boost regimen. BALB/c mice have a limited number of HIV-1 epitopes that can be used to evaluate the breadth of candidate vaccines thus this could not be evaluated in this study. Further studies using bi- and tri-valent HIV-1C mosaic immunogens to increase breadth, which is essential for clearing diverse strains of HIV-1 in infected individuals, will be carried out in non-human primates.

The stability and expression of transgenes is critical in recombinant BCG vaccine development. This is essential for memory cells to elicit a correct and potent immune response to the antigen in the event of an infection. The rBCGΔ*panCD* vaccines made in this study were stable *in vitro* and *in vivo*. The shuttle vectors in the BCG-Gag^M^ and BCG^E^ vaccines were detectable in peripheral lymphoid organs (spleen and lymph nodes) of vaccinated mice 11.5 weeks post vaccination. Furthermore, the *gag*^M^ DNA sequence obtained from BCG-Gag^M^ in the peripheral lymphoid organs was unaltered as determined by PCR and sequencing. This was encouraging, as these are sites where adaptive immune responses are initiated [53].

In order to make a stable rMVA an insertion site was selected that would be stable. In the past the del II and del III regions which lie within the variable terminal regions were used as sites of insertion in rMVA vaccine development. These regions are often prone to deletions and other mutational changes. Inserting a foreign gene into the variable terminal region makes it prone to such deletion mutations. Therefore to increase transgene stability, foreign genes have been inserted between transcriptionally convergent conserved genes where no possible transcriptional promoters could be disrupted [54,55]. In this study *gag*^*M*^ was inserted between the A11R and A12L genes. A Gag^M^ protein of the correct size (55kD) was shown to be expressed from MVA-Gag^M^ after vaccine scale up confirming the stability of the vaccine.

Live attenuated SIV and CMV vaccines that elicit persistent CD8^+^ T cell responses, have been shown to control viral load in macaques [56–58]. In this study, we demonstrated that BCG-Gag^M^ persists in the tissues of vaccinated mice as determined by the presence of BCG-Gag^M^ colonies in the peripheral lymphoid organs (spleen and lymph nodes) of these mice 11.5 weeks post vaccination (Fig 2). Our group and others have also shown that rBCG persists *in vivo* up to 20 weeks post vaccination [28,59–61]. Future studies of BCG-Gag^M^ should include experiments to confirm the long term persistence of HIV-specific T cell responses.

The replication of BCG *in vivo* is slow and rBCG persistence subsequently results in low antigen expression and low levels of antigen presentation [62]. Low T cell immune responses to the antigen are induced and differentiate into memory phenotype, and get stimulated when boosted with a matching antigen [63]. Vaccination with a BCG-Gag^M^ prime MVA-Gag^M^ boost generated predominantly effector memory cytokine-positive T cells, a T cell subset shown to play a role in the control of viral load after vaccination with CMV-based vaccines [56]. Effector memory cells act as the first line of defence at the site of HIV infection. Hansen and colleagues have shown that the protection of vaccinated non-human primates from SIV challenge was due to both CD4^+^ and CD8^+^ effector memory T cell responses [56–58].

Antigens derived from mycobacteria are processed and presented by macrophages. Antigens delivered into the phagolysosome usually get processed by the HLA class II pathway. Such antigens would induce CD4^+^ T cell responses (reviewed by Hess *et al*., 2000; [64]. In our study, the BCG-Gag^M^ prime and MVA-Gag^M^ boost resulted in the frequency of cytokine-secreting CD8^+^ T cells being greater than that of CD4^+^ T cells (Fig 5C and 5D). The Gag^M^ antigen in our study was linked to the 19kD signal sequence in the BCG shuttle vector. This targets the antigen to the cell wall, making it accessible for processing by the HLA class I pathway and inducing CD8^+^ T cell responses [65]. Previous studies carried out by our group also showed that the BCGΔ*pan* strain induced predominant CD8^+^ T cell responses to HIV-1 Gag [32,37,60]. CD4^+^ cells are essential for providing help to CD8^+^ T cells [66–69]. However, CD4^+^ T cells are also the target of HIV-1 infection (reviewed by Grossman et al., 2006 [70]). There is therefore a fine balance between inducing enough of a CD4^+^ response to provide help to CD8^+^ cells and inducing too many CD4^+^ cells, which will increase the pool of target cells for HIV-1 infection.

The Gag^M^ vaccines in our study induced a Th1 bias when administered in a heterologous or homologous prime-boost regimen (Fig 5E). A Th1 immune response has been shown to be important for protection against viral challenge in mice and humans as reported by Someya and colleagues (2004; [71]) and by Betts and colleagues respectively [72,73]. Both IFN-γ and TNF-α are important mediators of antiviral activity by CD8+ T cells in HIV-1 infection whilst increased production of IL-2 is associated with reduced viral loads in elite controllers [73–75]. It is therefore desirable for candidate vaccines to induce these cytokines as potential correlates of protection.

This study shows that subtype-specific monovalent HIV-1 Gag^M^ vaccines are highly immunogenic in mice. Furthermore, a low dose MVA-Gag^M^ boosted a BCG-Gag^M^ prime. This is very attractive for dose sparing and reduced costs for the targeted resource-limited regions should the vaccine get to clinical trials, licencing, and large scale distribution. This promising immunogenicity data warrants further evaluation in non-human primates.
